# Quantitative Ethylene Measurements with MOx Chemiresistive Sensors at Different Relative Air Humidities

**DOI:** 10.3390/s151128088

**Published:** 2015-11-06

**Authors:** Matic Krivec, Gerald Mc Gunnigle, Anže Abram, Dieter Maier, Roland Waldner, Johanna M. Gostner, Florian Überall, Raimund Leitner

**Affiliations:** 1 CTR Carinthian Tech Research AG, Europastrasse 12, A–9524 Villach, Austria; E-Mails: gerald.mcgunnigle@ctr.at (G.M.G.); raimund.leitner@ctr.at (R.L.); 2 Department for Nanostructured Materials, Jožef Stefan Institute, Jamova 39, SI–1000 Ljubljana, Slovenia; E-Mail: anze.abram@ijs.si; 3 Philips Consumer Lifestyle Klagenfurt, Koningsbergerstrasse 11, A–9020 Klagenfurt, Austria; E-Mails: dieter.maier@philips.com (D.M.); roland.waldner@philips.com (R.W.); 4 Division of Medical Biochemistry, Medical University of Innsbruck, Center for Chemistry and Biomedicine, Innrain 80, A–6020 Innsbruck, Austria; E-Mails: johanna.gostner@i-med.ac.at (J.M.G.); florian.ueberall@i-med.ac.at (F.Ü.)

**Keywords:** ethylene, relative humidity, chemiresistive sensor, linear regression model

## Abstract

The sensitivity of two commercial metal oxide (MOx) sensors to ethylene is tested at different relative humidities. One sensor (MiCS-5914) is based on tungsten oxide, the other (MQ-3) on tin oxide. Both sensors were found to be sensitive to ethylene concentrations down to 10 ppm. Both sensors have significant response times; however, the tungsten sensor is the faster one. Sensor models are developed that predict the concentration of ethylene given the sensor output and the relative humidity. The MQ-3 sensor model achieves an accuracy of ±9.2 ppm and the MiCS-5914 sensor model predicts concentration to ±7.0 ppm. Both sensors are more accurate for concentrations below 50 ppm, achieving ±6.7 ppm (MQ-3) and 5.7 ppm (MiCS-5914).

## 1. Introduction

Ethylene is a useful indicator of fruit ripeness and ethylene measurement systems are used commercially in the food industry [1]. Current systems require bulky near-infrared (NIR) laboratory equipment that limits their use to large warehouses. However, there is an economic incentive to monitor the fruit while it is in transit to improve its freshness, flavor, and shelf-life. This will require sensors that are compact, economic, and sensitive [2]. Furthermore, sensors must be able to account for the high relative humidities (up to 98%) encountered in this application. Metal oxide (MOx) sensors have been shown to be effective detectors of several volatile compounds; they are also small and economical [3]. Water vapors, however, act as a major disturbing factor in the sensing performance of these sensors. To improve the accuracy and reliability of this sensing method, compensating for the impact of water on MOx sensors is of a high significance [4]. Several compensation methods have been proposed so far: simple deduction of the humidity background value, three-dimensional approximation employing an artificial neuron network (ANN), and three-dimensional fit of the modified Clapeyron’s equation [4,5,6]. Although the existing methods showed relatively good results for the studied systems, they may not provide a universal solution for every situation (type of gas, sensing material, water concentration, *etc.*) This paper will investigate the suitability of two commercial MOx sensors for quantitative ethylene measurements at different relative air humidities.

The object of this work is a system that combines an ethylene sensor, a relative humidity (RH) sensor, and a model to predict the ethylene concentration from the two sensor readings. To achieve this we need to understand the mechanism of the ethylene sensors and the relationship between their responses at different levels of ethylene and relative humidity in their environment. To obtain an understanding of the structure and composition of the sensors we investigated both sensors with a scanning electron microscope (SEM). This revealed that the sensors have different active materials and radically different geometries. Differences in the microstructure of the sensing surfaces were also noted. To model the performance of the sensors we performed a series of experiments that measured the response of the sensors at different concentrations of ethylene and relative humidities. Different regression models were evaluated to predict the ethylene concentration from the sensor response and their performance was quantified.

SEM analysis of the devices showed that the MQ-3 is based on the Taguchi design [7] with a tin oxide sensing surface. The MiCS-5914 sensor is planar, with a tungsten oxide sensing surface and a smaller active area. Experimental measurements of the sensors’ responses confirmed that both sensors have an exponential time response as expected, though the tungsten sensor had smaller saturation and desaturation time constants (τ_s_ = 29 s, τ_d_ = 43 s) than the tin oxide sensor (τ_s_ = 80 s, τ_d_ = 165 s). Using measurements of relative humidity, the sensor response, and a linear regression model, the MQ-3 was able to predict ethylene concentration with an accuracy of ±6.7 ppm, with the MiCS-5914 sensor achieving ±5.7 ppm for concentration levels below 50 ppm.

## 2. Background

The mechanism by which a target gas affects the resistivity of a MOx device is still a matter of debate. The most popular hypothesis for n-type devices is that, in the absence of the target gas, atmospheric oxygen molecules are adsorbed on the sensing surface. The oxygen molecules strip the n-type material of its carriers, leaving a depletion layer with high resistivity. When a reducing gas, such as ethylene, comes into proximity with the sensing surface, it displaces the oxygen molecules, releasing the charge carriers and reducing resistivity [8,9]. This class of reaction can be modeled using the Langmuir-Hinshelwood equation, and several authors have expressed the response of a MOx sensor, *S*(*t*), in these terms:

(1) $$S\left(t\right)=S_{max}\frac{C_{voc}K}{1+C_{voc}K}\left(1-exp\left[-\frac{1+C_{voc}K}{K}kt\right]\right)$$

where $S_{max}$ is the maximum signal change when the sensor is fully saturated, $C_{voc}$ is the concentration of volatile organic compound (VOC) in gaseous phase, K is the adsorption equilibrium constant of the VOC, and k is the forward rate constant [10].

The sensitivity of a MOx sensor is affected by several factors: its chemical composition, surface modification, and the microstructure of the sensing surface, as well as the temperature and humidity of its environment. Environmental humidity strongly influences the performance of MOx gas sensors and, in fact, similar sensors have been developed specifically to detect water vapor. Humidity affects the sensor in a similar way as a volatile gas. This will reduce baseline resistance because of the reduced surface area for the adsorption of O_2_ and VOC molecules due to the competitive physisorption and chemisorption of water molecules [11,12]. Water vapor reduces the sensitivity of both sensors to ethylene gas because water molecules compete with ethylene molecules for oxygen species and reduce surface area available for the adsorption of ethylene molecules on the MOx surface [10,11].

The response of a MOx sensor to ethylene gas at different relative humidities can be described as a two-molecular Langmuir-Hinshelwood system, where both types of molecules (ethylene and water) compete for the same adsorption sites on the MOx surface. The model developed in this paper assumes that adsorption and desorption are in equilibrium and that the sensor response, S(t), has stabilized ($\underset{t\to \infty }{\lim}\frac{dS}{dt}=0$). The time-dependent part is no longer relevant and Equation (1) is re-written as:

(2) $$S_{C2H4}=S_{max,C2H4}\frac{C_{C2H4}K_{C2H4}}{1+C_{C2H4}K_{C2H4}+C_{H2O}K_{H2O}}$$

where $S_{C2H4}$ is the sensor response at the steady state, $S_{max,C2H4}$ is the maximum signal change when the sensor is fully saturated, $C_{C2H4}$ and $C_{H2O}$ are the concentrations of ethylene and water vapors in gaseous phase, $K_{C2H4}$ and $K_{H2O}$ are the adsorption equilibrium constants of ethylene and water molecules. Equation (2) derived from Langmuir-Hinshelwood would motivate a non-linear regression model to predict the ethylene concentration from the sensor response (and humidity). Non-linear models suffer from over-fitting and require a lot of measurement data for stable parameter estimation. We tried to find a linear regression model that achieves a good and stable accuracy. The results support that the prediction performance of the linear regression model for the ethylene concentration is better than the non-linear model.

## 3. Experimental Section

### 3.1. Scanning Electron Microscope (SEM) Characterization

The structural characterization of the functional parts and the MOx-sensitive layer of the MQ-3 and MiCS-5914 sensors was conducted using a scanning electron microscope (SEM; Jeol JSM-7600F) equipped with a field-emission gun. The microscope was operated between 5 and 15 kV. The chemical composition was measured using an energy-dispersive X-ray spectrometer (EDS; INCA Oxford 350).

### 3.2. Gas Sensitivity Measurements

**Figure 1:**
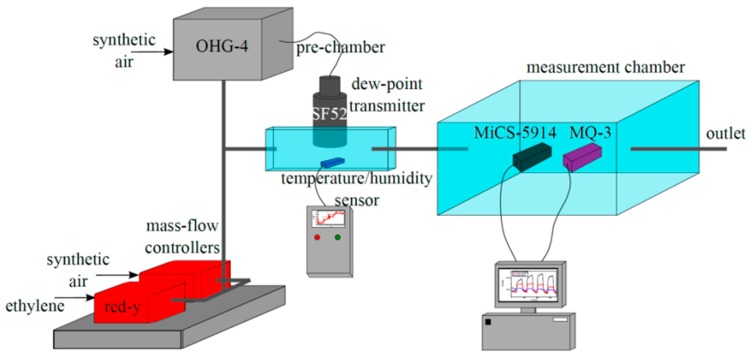
Schematic diagram of the gas measurement setup.

The experimental setup is shown in Figure 1. A controlled mixture of ethylene and synthetic air is combined with synthetic air at a specified humidity. The resulting gas flows first to a pre-chamber, in which temperature, humidity, and dew-point are measured, then to the main measurement chamber before being vented in the atmosphere.

The pre-chamber (V = 0.05 L) holds the dew-point transducer (SF52, Michell Instruments, Cambridge, UK) and the temperature/humidity sensor (SHT31-ARP, Sensirion AG, Staefa, Switzerland). Two MOx-based gas sensors, *i.e*., MQ-3 (Henan Hanwei Electronics Co., Ltd., Zhengzhou, China) and MiCS-5914 (SGX Sensortech SA, Corcelles, Switzerland), were positioned inside the stainless steel measurement chamber (V = 0.17 L). Both gas sensors contain:

- a MOx-sensing layer (SnO_2_ and WO_3_ functional layer),
- an integrated heater to be connected to a 5 V power supply,
- two read-out contacts. The resistivity of the MQ-3 sensor is measured directly. The MiCS-5914 sensor and an external resistor form a voltage divider; the voltage across the external resistor is taken as the output.

The chambers were connected to an inlet pipeline regulated by two mass-flow controllers (red-y, Vögtlin Instruments AG, Aesch, Switzerland) dosing synthetic air and ethylene gas diluted in synthetic air (200 ppm), and a humidity generator (OHG-4 Humidity Generator, Owlstone Ltd., Cambridge, UK).

Measurements were conducted under continuous air flow (500 mL/min) at four different ethylene concentrations (50, 40, 25, and 10 ppm) and three relative humidities (0%, 33%, and 64%). Both sensors were first stabilized by flushing the gas chamber with synthetic air for 1200 s. Then, an ethylene air mixture was introduced to the gas chamber for a period of 600 s before being flushed. This was repeated for various ethylene concentrations. For the MiCS-5914 sensor, additional measurements were made with a with longer stabilization time (4500 s) at 0%, 33%, 50%, and 64% relative humidity.

### 3.3. Development of an Ethylene Prediction Model as a Function of Relative Humidity

For each relative humidity, the ethylene concentration of the chamber was ramped up to the specified value and then the chamber was flushed. The measurement quantity, the voltage for the MiCS-5914 sensor and the resistance for the MQ-3, was measured as a function of time. The resulting signal was smoothed with a five-point moving average filter. The sensor baseline was measured in the middle of the flush phase between the 25 ppm and 40 ppm concentration phases. Sample points were chosen at points just before flushing, *i.e*., when the ethylene concentration had, presumably, attained equilibrium. These values are plotted against the known concentrations, along with a least squares (LS) linear regression, for the different values of relative humidity (Figures S1–S3). A more comprehensive model that is a linear function of the concentration, relative humidity, and their product has been developed.

## 4. Results and Discussion

### 4.1. SEM Characterization of the Sensors

**Figure 2:**
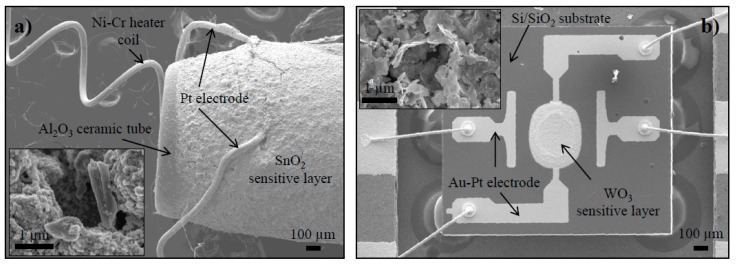
SEM micrograph of the functional parts in (**a**) MQ-3 and (**b**) MiCS-5914 sensor (insets: sensitive MOx layer at higher magnification).

The MQ-3 sensor, shown in Figure 2a, is based on the Taguchi design. A thick MOx film is deposited around the alumina ceramic tube with platinum electrode wires on top and a coiled Ni-Cr heating element inside the tube. The energy dispersive measurements (Figure S4) show the presence of a tin oxide sensing layer with traces of zinc (possibly in the form of zinc oxide or alternatively acting as a dopant). The sensing layer is porous with small SnO_2_ nanoparticles (approximately 50 nm in diameter) forming larger agglomerates.

The MiCS-5914 sensor, shown in Figure 2b, has a more sophisticated design. The device has a Si/SiO_2_ substrate on which four gold-platinum electrodes (two for measurement and two for heating) and the MOx sensing layer (450 × 550 µm) are deposited. Closer inspection of the sensing layer (inset of Figure 2b) shows it to be composed of tungsten nano-plates approximately 100 nm wide and 20 nm thick. Traces of tin were detected (Figure S5), either as an oxide or as a dopant.

### 4.2. Qualitative Description of the Sensor Responses

In the background section we modeled the sensors with the Langmuir-Hinshelwood (LH) model. This describes the response of the sensor to ethylene, the competitive effect of water vapor, and the time behavior of the sensor. In this sub-section we will examine the relationship of the sensorsʼ response with ethylene and the sensorsʼ transient behavior. Both of these characteristics are affected by humidity and cannot be examined in isolation. However, for clarity, in this sub-section we will consider only the qualitative effect of the humidity; a quantitative analysis is postponed to Section 4.4.

**Figure 3:**
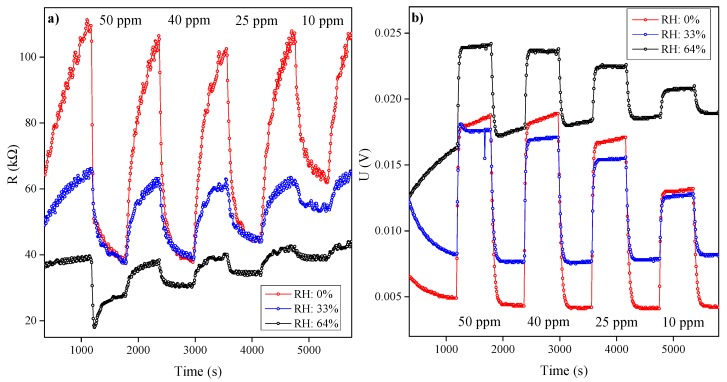
Response and recovery measurements of (**a**) MQ-3 sensor measured in the resistivity mode; and (**b**) MiCS-5914 sensor measured in the voltage mode, at different relative humidities.

The response of the MQ-3 sensor is shown in Figure 3a. As predicted in the background, the resistivity of this n-type semiconductor falls as the ethylene concentration rises. The effect is most pronounced at low humidities; increasing humidity increases the competitive effect of water molecules and reduces the sensor’s sensitivity to ethylene. However, even at 64% RH, ethylene concentrations of 10 ppm are discernible. The sensor’s transient response exhibits the exponential behavior predicted by the LH equation. However, the reaction at the tin oxide surface is slow with relatively long sorption and desorption time constants (τ_s_ = 80 s, τ_d_ = 165 s).

Figure 3b shows the results obtained with the MiCS-5914 sensor. The actual measurement is the voltage across an external resistor that, together with the sensor, forms a voltage divider. The external resistor has a smaller resistance than the sensor, and the measured voltage (U) varies with:

(3) $$U=\;U_{0}\left(\frac{R_{L}}{R_{MOx}+\;R_{L}}\right)\;≅\;U_{0}\left(\frac{R_{L}}{R_{MOx}}\right)$$

where $U_{0}$ is the supply voltage (5 V), $R_{L}$ is the resistance of an external resistor, and $R_{MOx}$ is the resistance of the MOx layer. As with the MQ-3 sensor, resistance falls as the ethylene concentration rises. Rising humidity reduces the effect, but also reduces the baseline resistance (*i.e*., the resistance at 0% concentration). Like the previous sensor, the MiCS-5914 sensor also has an exponential time behavior, though the response is much more rapid (τ_s_ = 29 s, τ_d_ = 43 s). However, at high humidities, a slow exponential trend is apparent in the baseline; to reduce the influence of this effect we repeated this experiment over a longer timescale.

### 4.3. Response of the Sensors to Ethylene and Humidity

**Figure 4:**
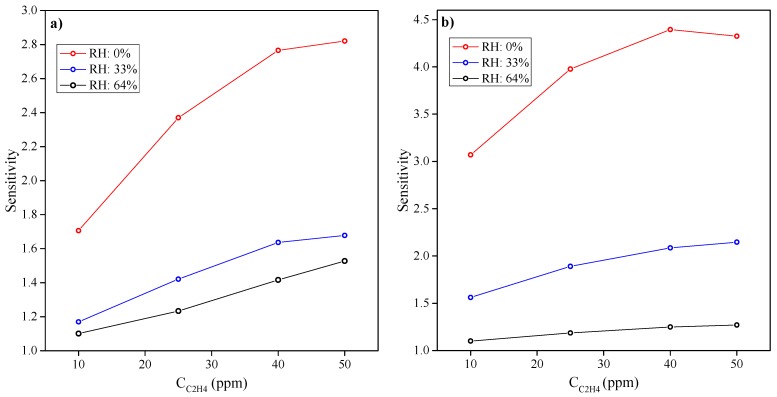
Sensitivities of the (**a**) MQ-3 sensor and (**b**) MiCS-5914 sensor to different ethylene concentrations at different relative humidities.

Figure 4 shows the relationship between the sensor sensitivity and ethylene concentrations at different relative humidities. The sensitivity was calculated by normalizing a given signal at a distinct ethylene concentrations to the baseline signal at an individual RH. For a given humidity, both sensors show a monotonic, near-linear response to rising ethylene. Increasing the level of humidity reduces sensitivity, though it does make the ethylene response more linear.

The MiCS-5914 sensor is far more sensitive to ethylene gas than the MQ-3 sensor at low RH (0% and 33%), though both sensors have similar sensitivities at 64% RH. The lower sensitivity of the MQ-3 sensor is rather surprising because our previous work found that SnO_2_ is more sensitive than WO_3_ to ethylene gas [13].

We hypothesize that the MiCS-5914 sensor responds more quickly to changes in ethylene concentration because of the thermal properties of the sensing surface. The sensing layer is much smaller than that of the MQ-3, allowing it to be heated more homogeneously and allowing the surface to be compensated more rapidly for changes in temperature induced by the gas mixture. In contrast, the MQ-3 uses a coiled Ni-Cr wire inside the ceramic tube to heat the sensing surface. We suggest this will heat the surface much less homogeneously because not all coils are in contact with the ceramic. As a consequence, heating is more localized and a corrective response to external changes is slower.

Both sensor responses correlate well with ethylene concentration from 10 ppm to 40 ppm (R^2^ > 0.97 for MQ-3 and R^2^ > 0.95 for MiCS-5914 sensor), and this is independent of humidity. However, the evaluation of ethylene concentrations higher than 40 ppm would be difficult, especially at low humidities.

### 4.4. Ethylene Prediction Model as a Function of Relative Humidity

In the previous subsection we modeled sensor response as a function of humidity and ethylene concentration; in this section we invert the model to estimate ethylene concentration from the sensor response. Both sensor responses (Figure 4) exhibit a non-linear behavior as predicted by the Langmuir-Hinshelwood model. To predict the ethylene concentration from the MOx sensor and humidity reading, we evaluate a linear regression model extended with a cross-term in Equation (4). Separate linear models for constant humidity gave promising results (Figures S1 and S2). The cross-term $\beta_{3}S_{C2H4}S_{H2O}$ is necessary to let the model adapt for the reduced sensitivity with increasing relative humidity. Equation (4) describes the used ethylene prediction model.

(4) $${\vec{x}}_{i}=\left(S_{C2H4},S_{H2O}\right)\;{\overset{ˇ}{C}}_{C2H4}=\bar{f\left({\vec{x}}_{i},\vec{\beta }\right)}=\beta_{0}+\beta_{1}S_{C2H4}+\beta_{2}S_{H2O}+\beta_{3}S_{C2H4}S_{H2O}\;\vec{\overset{ˇ}{\beta }}=argmin||C_{C2H4}-f{\left({\vec{x}}_{i},\vec{\beta })|\right|}_{2}^{2}$$

where $\vec{\beta }$ is the regression model, $\vec{\overset{ˇ}{\beta }}$ is the LS solution, $S_{C2H4}$ is the MOx sensor reading, $S_{H2O}$ is the humidity sensor reading, $C_{C2H4}$ is the measured ethylene concentration, and ${\overset{ˇ}{C}}_{C2H4}$ is the predicted ethylene concentration.

**Figure 5:**
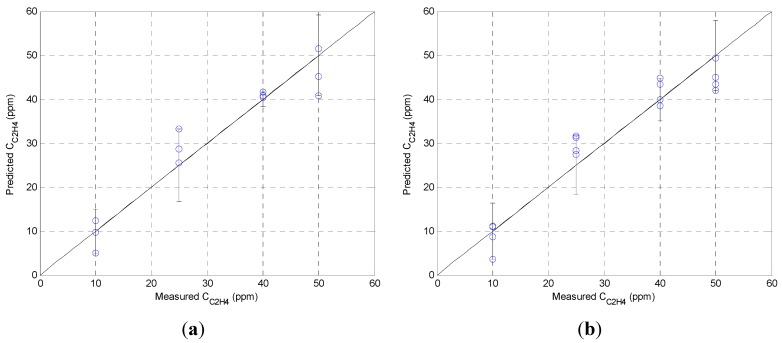
The combined prediction model as a linear function of the ethylene concentration, relative humidity, and their product for the (**a**) MQ-3 and (**b**) MiCS-5914 sensors.

Figure 5 shows the prediction for the ethylene concentration based on the model, the sensor reading, and the relative humidity. For the MQ-3 sensor the prediction accuracy (σ_max_) is ±9.2 ppm (Figure 5a), and it is ±7.9 ppm for the MiCS-5914 sensor (Figure 5b). We plan to extend the ethylene concentration range; however, currently this is limited by our experimental setup. The MQ-3 sensor is more accurate below 50 ppm (±6.7 ppm).

**Figure 6:**
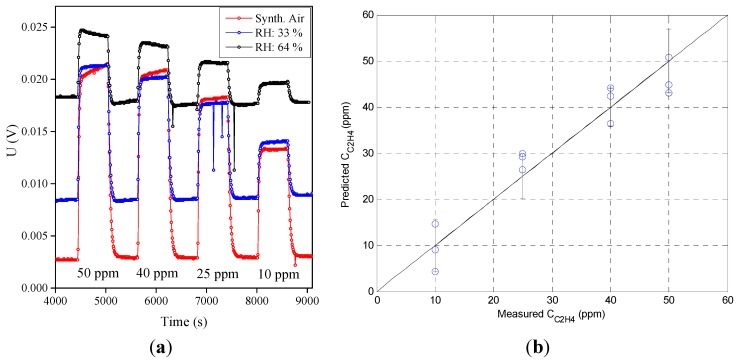
(**a**) Response and recovery measurements of MiCS-5914 sensor with longer stabilization (4500 s) at different relative humidity; (**b**) The corresponding combined prediction model as a linear function of the concentration, relative humidity, and their product.

We repeated the experiment with longer stabilization times using the MiCS-5914 sensor. The response and recovery measurements in Figure 6a show that the drift of the background level, seen at 64% relative humidity in Figure 3b, is now no longer present. Using this data, the prediction model in Figure 6b shows an improved accuracy of ±7.0 ppm and ±5.7 ppm for concentrations equal and lower to 50 ppm.

## 5. Conclusions

The sensitivity of two MOx-based chemiresistive sensors, MQ-3 and MiCS-5914, to ethylene gas at different relative air humidities has been investigated. The MiCS-5914 sensor responded more quickly to changes in ethylene concentration, having much smaller saturation and desaturation time constants (τ_s_ = 29 s, τ_d_ = 43 s) than the MQ-3 sensor (τ_s_ = 80 s, τ_d_ = 165 s). Relative humidity had a similar effect on both sensors, significantly reducing their sensitivity to ethylene. Models that predict ethylene concentration as a function of the sensor reading and the relative humidity were developed and evaluated for each sensor. The models achieved an accuracy of ±9.2 ppm for the MQ-3 sensor and ±7.0 ppm for the MiCS-5914 sensor. More accurate predictions can be obtained for lower ethylene concentrations (below 50 ppm), with ±6.7 ppm for the MQ-3 and ±5.7 ppm for MiCS-5914 sensor.

## Supplementary Materials

Supplementary materials can be accessed at: http://www.mdpi.com/1424-8220/15/11/28088/s1.
