# Supplementary material for: Quantitative Ethylene Measurements with MOx Chemiresistive Sensors at Different Relative Air Humidities
Source: Sensors (Basel). 2015 Nov 6;15(11):28088–98. doi: 10.3390/s151128088 (PMC4701270; doi:10.3390/s151128088)
Supplement: Supplementary file 1 [file sensors-15-28088-s001.pdf]

OPEN ACCESS

sensors

ISSN 1424-8220

www.mdpi.com/journal/sensors

Supplementary Information

Quantitative Ethylene Measurements with MOx Chemiresistive Sensors at Different Relative Humidities. *Sensors* 2015, *15*, 28088-28098

Matic Krivec [1,†,*], Gerald Mc Gunnigle [1,†], Anže Abram [2], Dieter Maier [3], Roland Waldner [3], Johanna M. Gostner [4], Florian Überall [4] and Raimund Leitner [1,†]

1. CTR Carinthian Tech Research AG, Europastrasse 12, A–9524 Villach, Austria; E-Mails: gerald.mcgunnigle@ctr.at (G.M.G.); raimund.leitner@ctr.at (R.L.)
2. Department for Nanostructured Materials, Jožef Stefan Institute, Jamova 39, SI–1000 Ljubljana, Slovenia; E-Mail: anze.abram@ijs.si
3. Philips Consumer Lifestyle Klagenfurt, Koningsbergerstrasse 11, A–9020 Klagenfurt, Austria; E-Mails: dieter.maier@philips.com (D.M.); roland.waldner@philips.com (R.W.)
4. Division of Medical Biochemistry, Medical University of Innsbruck, Center for Chemistry and Biomedicine, Innrain 80, A–6020 Innsbruck, Austria; E-Mails: johanna.gostner@i-med.ac.at (J.M.G.); florian.ueberall@i-med.ac.at (F.Ü.)

† These authors contributed equally to this work.

* Author to whom correspondence should be addressed; E-Mail: matic.krivec@ctr.at; Tel.: +43-4242-56300-273; Fax: +43-4242-56300-400.

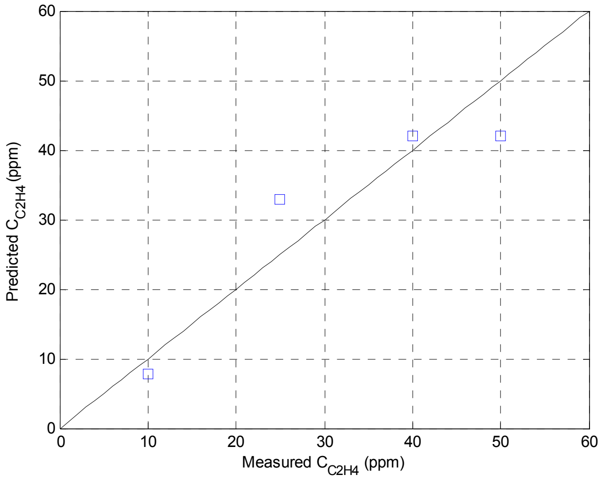

(**a**)

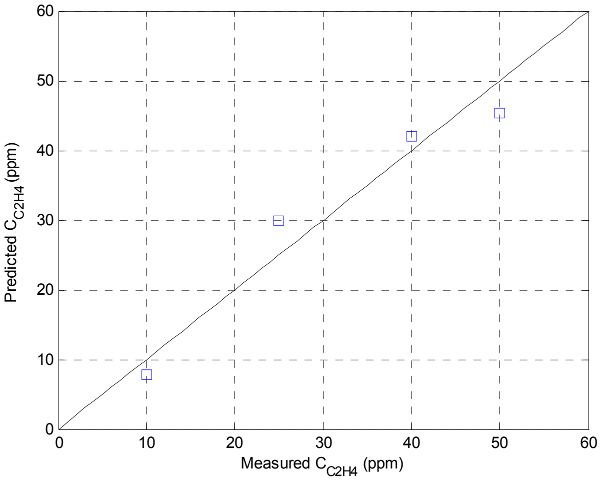

(**b**)

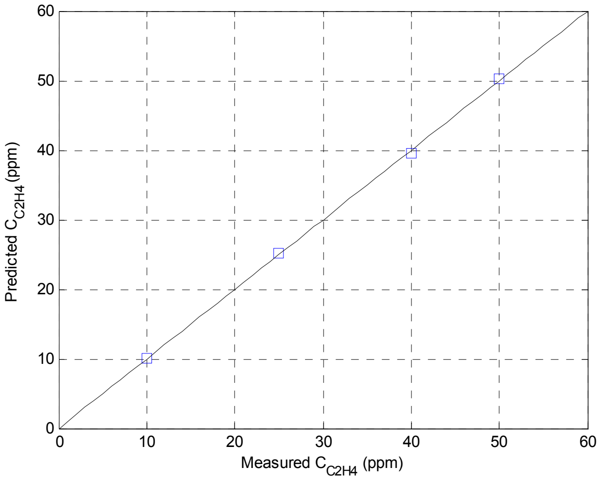

(**c**)

Figure S1. The correlation between the measured and predicted ethylene concentration at (**a**) 0%; (**b**) 33%; and (**c**) 64% relative humidity for MQ-3 sensor.

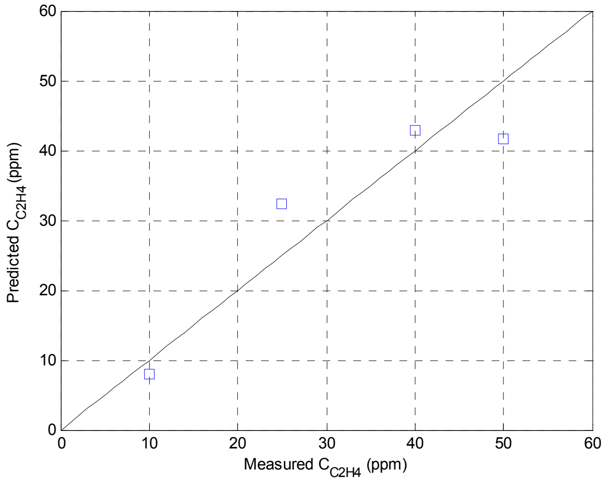

(**a**)

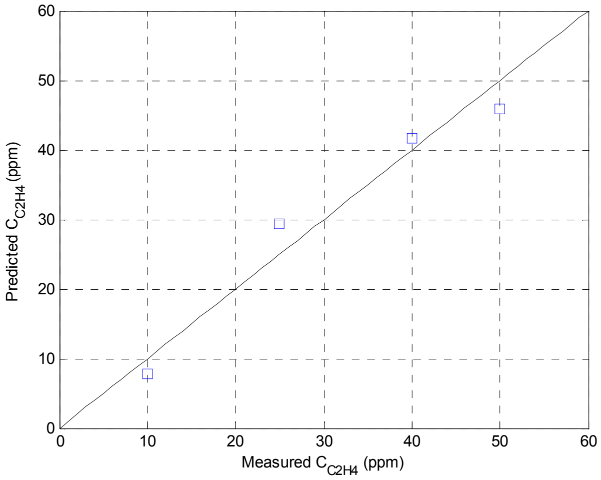

(**b**)

Figure S2. *Cont.*

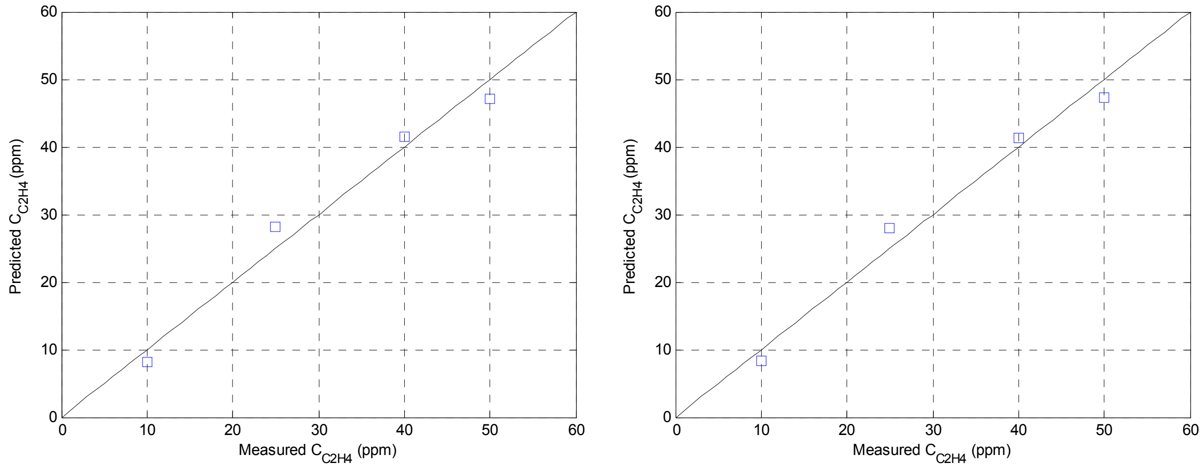

(c) **(d)**

Figure S2. The correlation between the measured and predicted ethylene concentration at (**a**) 0%; (**b**) 33%; (**c**) 50%; and (**d**) 64% relative humidity for MiCS-5914 sensor.

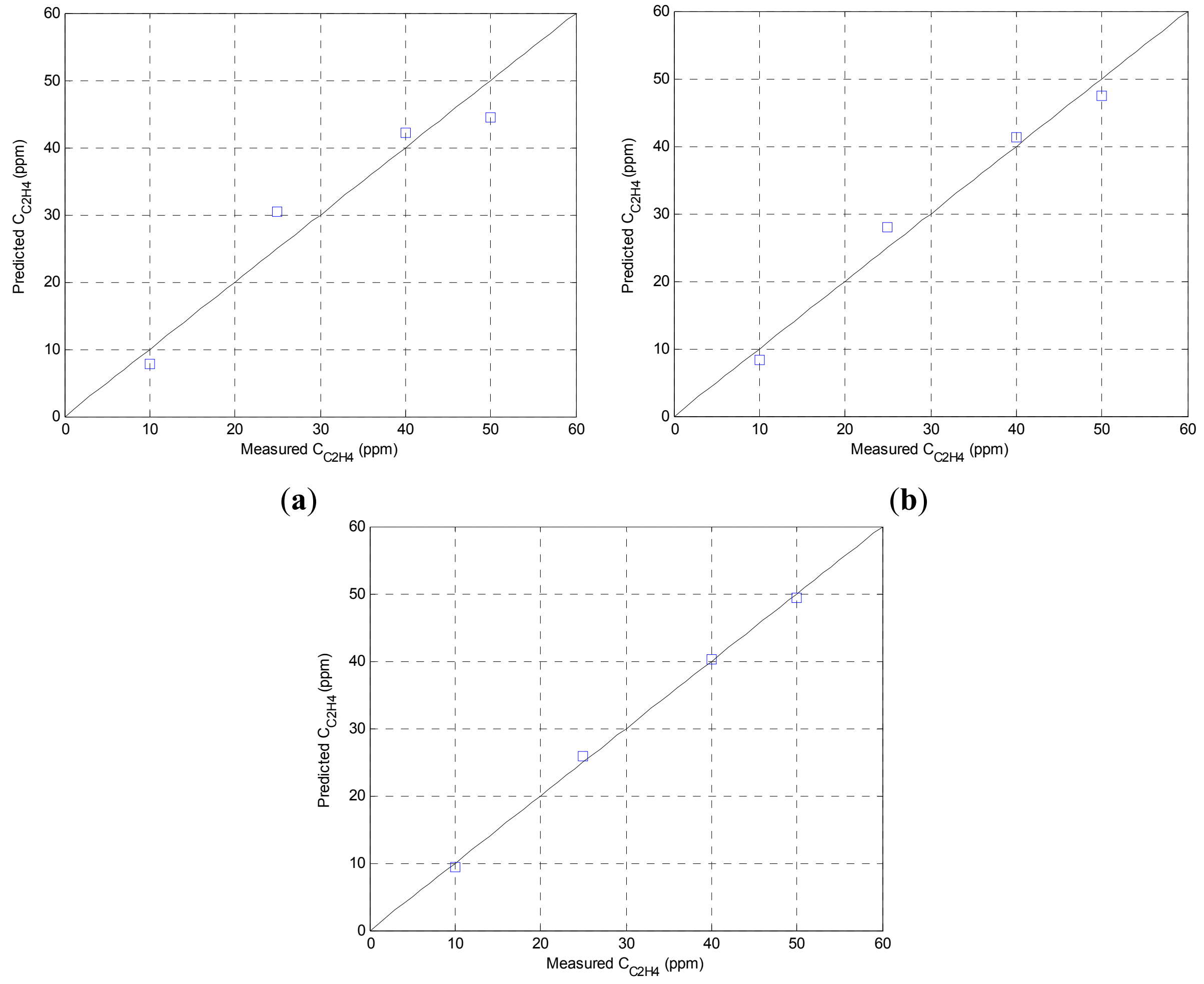

(a) **(b)**

(c)

Figure S3. The correlation between the measured and predicted ethylene concentration at (**a**) 0%; (**b**) 33%; (**c**) 50% and (**d**) 64 % relative humidity for MiCS-5914 sensor. Measurements were conducted using longer stabilisation time (4500 s).

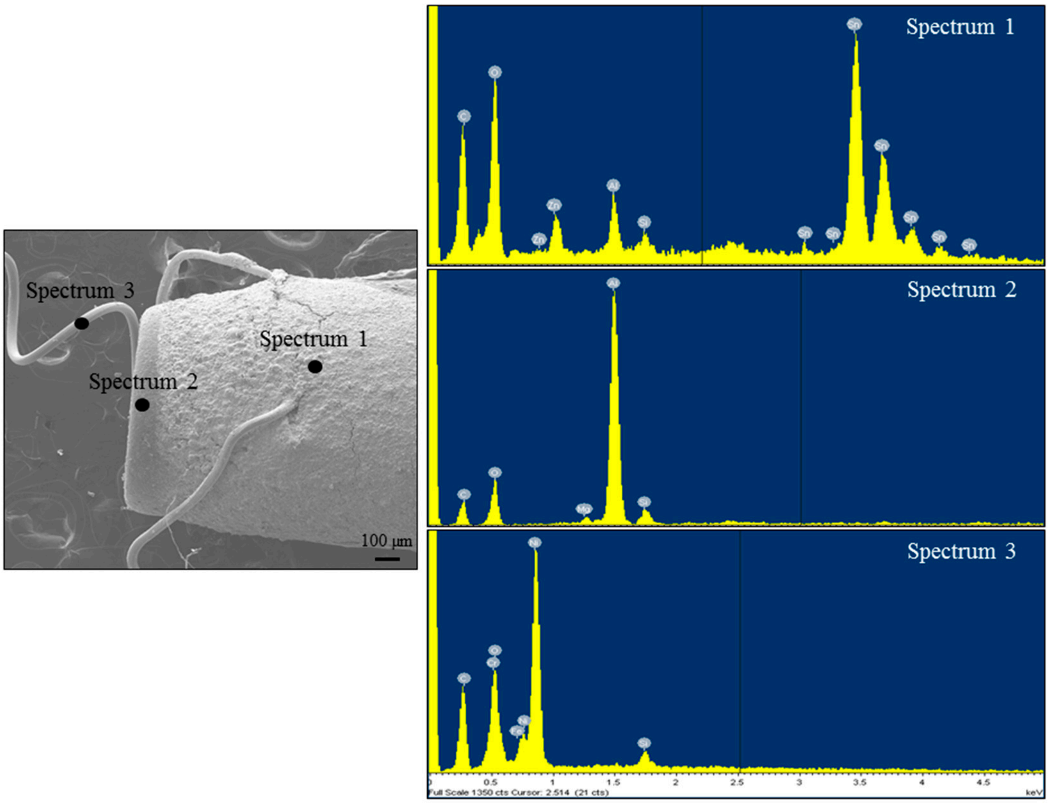

Figure S4. Scanning electron microscope (SEM) and energy dispersive spectroscopy (EDS) analysis of the main functional parts of the MQ-3 sensor.

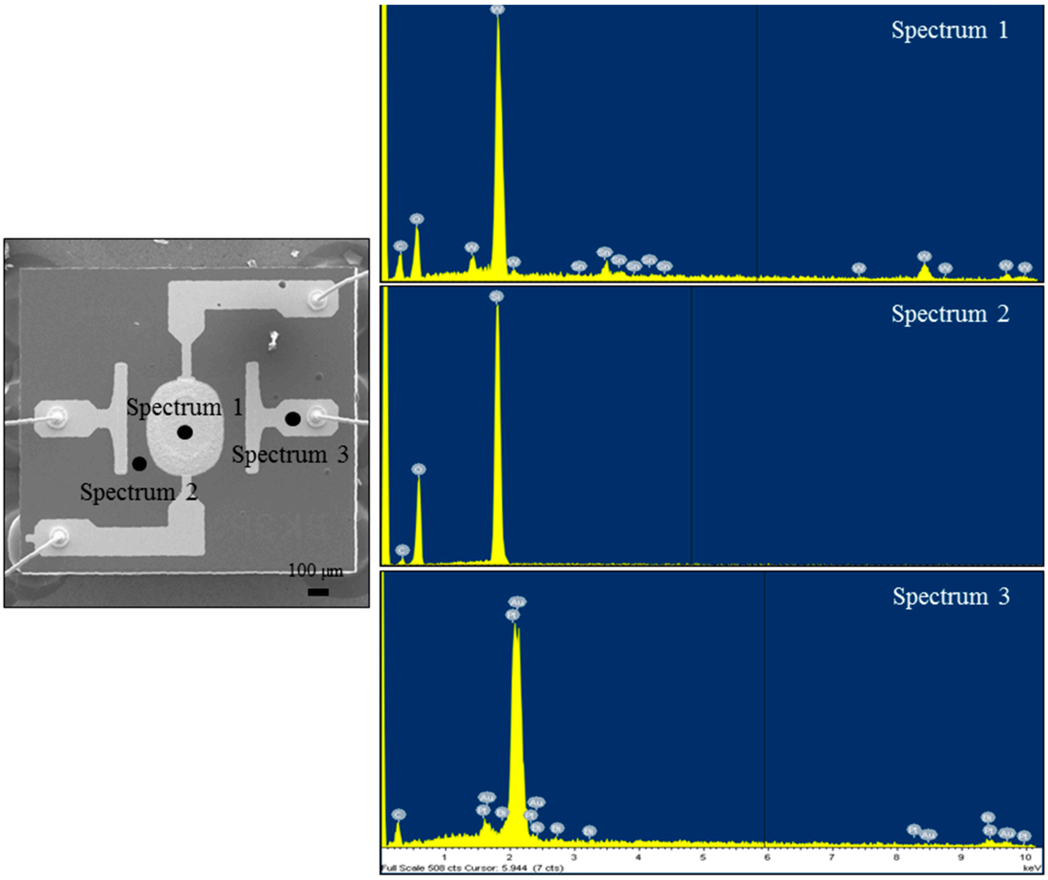

Figure S5. SEM and EDS analysis of the main functional parts of the MiCS-5914 sensor.
